# Lessons from Comparison of Hypoxia Signaling in Plants and Mammals

**DOI:** 10.3390/plants10050993

**Published:** 2021-05-17

**Authors:** Catherine M. Doorly, Emmanuelle Graciet

**Affiliations:** 1 Department of Biology, Maynooth University, W23 F2K6 Maynooth, Ireland; CATHERINE.DOORLY.2020@MUMAIL.IE; 2 Kathleen Lonsdale Institute for Human Health Research, Maynooth University, W23 F2K8 Maynooth, Ireland

**Keywords:** hypoxia, plants, mammals, ubiquitin/proteasome system, SUMO, N-degron pathway, nitric oxide

## Abstract

Hypoxia is an important stress for organisms, including plants and mammals. In plants, hypoxia can be the consequence of flooding and causes important crop losses worldwide. In mammals, hypoxia stress may be the result of pathological conditions. Understanding the regulation of responses to hypoxia offers insights into novel approaches for crop improvement, particularly for the development of flooding-tolerant crops and for producing better therapeutics for hypoxia-related diseases such as inflammation and cancer. Despite their evolutionary distance, plants and mammals deploy strikingly similar mechanisms to sense and respond to the different aspects of hypoxia-related stress, including low oxygen levels and the resulting energy crisis, nutrient depletion, and oxidative stress. Over the last two decades, the ubiquitin/proteasome system and the ubiquitin-like protein SUMO have been identified as key regulators that act in concert to regulate core aspects of responses to hypoxia in plants and mammals. Here, we review ubiquitin and SUMO-dependent mechanisms underlying the regulation of hypoxia response in plants and mammals. By comparing and contrasting these mechanisms in plants and mammals, this review seeks to pinpoint conceptually similar mechanisms but also highlight future avenues of research at the junction between different fields of research.

## 1. Introduction

With the evolution of photosynthetic organisms, cells adapted to the resulting increase in oxygen in the atmosphere. Living organisms developed mechanisms to sense oxygen levels and respond to fluctuations while also becoming reliant on oxygen for biochemical reactions or as the final electron acceptor in the mitochondrial electron transport chain to generate energy for cellular functions (reviewed in [1,2]). As dependence on oxygen evolved, hypoxia (or reduced oxygen availability) became a stress that can affect survival if prolonged. For example, in plants, including in many staple crops, hypoxia reduces growth and productivity and can negatively affect the responses to other stresses [3]. Both plants and mammals experience hypoxia and its negative effects. Notably, both have evolved conceptually similar molecular mechanisms to sense and respond to hypoxia stress, making it interesting to draw parallels between hypoxia-related signaling and responses in plants and mammals (see also [1,2]).

Hypoxia can occur as a result of a physiological or developmental state (this type of hypoxia has been coined ‘chronic hypoxia’; terminology reviewed in [4]), which is often the result of limited oxygen availability due to the diffusion distance between source and cells within a tissue or organ. For example, in plants, meristems, roots, or seeds (as well as other tissues) are known to have oxygen levels below 5% [5,6,7]. In mammals, tissues with low vascular density will experience chronic hypoxia, and similarly to plants, low oxygen conditions contribute to the maintenance of stem cells [5,8]. In contrast, hypoxia-related stress (also sometimes termed ‘acute hypoxia’) stems from a decrease in oxygen availability. Examples of such hypoxia-related stress in plants include the effects of soil waterlogging or plant submergence underwater. Indeed, due to the limited diffusion of oxygen in water, flooding conditions reduce oxygen availability in roots but also in shoots upon submergence. The latter also reduces light and carbon dioxide availability, thus also impacting photosynthesis and carbohydrate availability (reviewed in [9,10]). In mammals, such acute hypoxia stress can occur in the context of solid tumors, ischemia, or hypoxic injury (e.g., as a consequence of heart attack), for example [11]. In both plants and mammals, the impact of hypoxia manifests as an energy crisis, nutrient and carbohydrate depletion, and oxidative stress, thus highlighting the complex nature of this stress and the challenges in understanding signaling pathways and the onset of adaptative responses.

Under normoxia, oxidative phosphorylation is the primary pathway through which aerobic organisms generate cellular ATP. In contrast, under hypoxic conditions, less oxygen is available to maintain oxidative phosphorylation, and cells instead rely on glycolysis for ATP production and on fermentative pathways to regenerate NAD+ [9,10,12,13]. However, if hypoxia persists, decreased sugar level availability can lead to carbon starvation, thus triggering additional changes, such as reduced energy consumption and an increase in catabolic metabolism [12]. Hypoxia may therefore be sensed via different mechanisms and pathways, such as oxygen sensing, energy sensing, and/or detection of nutrient and sugar depletion/starvation. Notably, these are common features of hypoxia sensing at the cellular level in both plants and mammals (reviewed in detail in [13]).

Hypoxia stress in plants and mammals also induces the production of highly reactive signaling molecules, including reactive oxygen species (ROS) and nitric oxide (NO) [13,14,15,16], both of which act in concert with each other, largely through the (reversible or irreversible) modification of regulatory proteins, such as those involved in signal transduction cascades (e.g., kinases, phosphatases, and transcription factors) [16]. S-nitrosylation of cysteine residues is an important reversible protein modification that contributes to mediating the effects of NO and regulates protein activity (e.g., activating the substrate protein or targeting it for degradation) (reviewed in [15,17]). Similarly, ROS can trigger a range of post-translational modifications that regulate the activity of target proteins (e.g., disulfide bridge formation, S-glutathionylation, sulfhydration, etc.) [15,18]. Notably, ROS not only alter proteins, but also lipids, polysaccharides, and nucleic acids, which can lead to irreversible damage if uncontrolled [13,14,15]. Hence, while the production of ROS and NO is essential for the onset of hypoxia response, their degradation via the activation of anti-oxidant mechanisms is equally important to avoid cell damage and ensure cell homeostasis.

The onset of responses to hypoxia requires a reprogramming of the genome. The latter relies not only on the activity of master transcriptional regulators that orchestrate gene expression in response to hypoxia but also important epigenetic changes and the control of translation [13,19,20]. Key aspects of hypoxia sensing and response also rely on the fine-tuning of key regulatory genes and proteins. In both plants and mammals, protein degradation mediated by the ubiquitin/proteasome system is central to this regulation. In recent years, the ubiquitin-like protein SUMO (Small Ubiquitin-like Modifier) has also been shown to play an important role in the regulation of hypoxia response and sensing mechanisms in plants and mammals [21,22,23,24]. For example, the complement of sumoylated proteins changes upon hypoxia in mammalian cells, with an overall increase in sumoylated proteins [25]. In comparison to mammals, much less is known about the role of SUMO in the regulation of hypoxia responses in plants, thus making it particularly interesting to draw comparisons, especially since SUMO’s role in plant responses to several other abiotic and biotic stress is now coming to light (e.g., [26,27,28,29,30,31,32,33,34,35]; also reviewed in [22,36,37]).

A similar cascade of reactions is involved in the conjugation of ubiquitin and SUMO to target proteins, including E1 activating, E2 conjugating, and E3 ligase-type proteins (reviewed in [22,38,39]). Ubiquitin and SUMO also have a conserved C-terminal glycine that is crucial for their conjugation to target proteins via the formation of an isopeptide bond between this last glycine residue and the ε amino group of a lysine residue on the target protein (or on a previously conjugated ubiquitin or SUMO in the case of poly-ubiquitin or poly-SUMO chain formation, respectively) (reviewed in [22,38,39]). Notably, SUMO is also involved in modulating the stability of its targets through promoting or preventing their degradation by the ubiquitin/proteasome system, thus highlighting the crosstalk(s) between the ubiquitin and SUMO systems [40,41]. SUMO also mediates changes in the subcellular localization, activity, and protein/protein interactions of its target proteins [36,37]. Together with the reversible nature of ubiquitination and sumoylation due to the activity of specific proteases [25,36,42,43,44], these two post-translational modifications contribute to rapidly changing the protein landscape and govern the fast and fine-tuning of the hypoxia response.

Here, we review the roles of ubiquitin and SUMO in the regulation of hypoxia sensing and response in both plants and mammals, with a focus on cellular responses that are relatively well conserved in both kingdoms in order to draw parallels and highlight gaps of knowledge.

## 2. Ubiquitin and Sumoylation Function in Oxygen Sensing and in Downstream Signal Transduction

**Figure 1:**
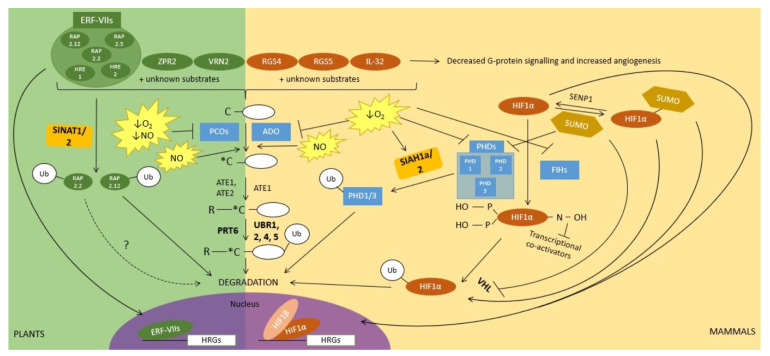
Regulation of oxygen sensing and downstream signals in plants and mammals, with a focus on ubiquitination and sumoylation. Oxygen sensing is mediated by oxygen-dependent enzymes (PCOs in plants, and ADO, PHDs, and FIHs in mammals) that regulate cellular responses to oxygen (O_2_) levels. These enzymes contribute to the regulation of the stability of transcription factors that act as master regulators of hypoxia response genes (i.e., ERF-VIIs in plants and HIF1α in mammals). Oxidation of N-terminal cysteine residues by PCOs and ADO (in plants and mammals, respectively) results in the degradation of target proteins *via* the evolutionarily conserved N-degron pathway. In plants, this includes the ERF-VII transcription factors, following their arginylation by ATE enzymes and ubiquitination by the E3 ubiquitin ligase PRT6. In mammals, PHDs and FIHs hydroxylate specific proline and asparagine residues, respectively, on HIF1α, which can then be ubiquitinated by the E3 ubiquitin ligase VHL. A conserved group of E3 ubiquitin ligases, SINAT1/2 in plants and SIAH1a/2 in mammals, also regulate the stability of hypoxia master regulators in plants and mammals. Sumoylation is involved in the regulation of HIF1α; however, there are conflicting reports on its effect on HIF1α.

Oxygen-sensing mechanisms in both plants and animals rely on the activity of oxygen-dependent enzymes that post-translationally modify master regulators of hypoxia response when oxygen is available. While these enzymes and their substrates differ in plants and animals, the downstream effects are conceptually and functionally strikingly similar. Indeed, in both plants and mammals, the master regulators of hypoxia response are transcription factors that are (i) modified by oxygen-dependent enzymes; (ii) rapidly degraded in normoxia, but instead stabilized under hypoxic conditions, thus resulting in their accumulation and in the subsequent activation of the hypoxia response program (Figure 1). For an evolutionary perspective on the evolution of oxygen sensing mechanisms, we refer the readers to these two excellent reviews [1,2].

In plants, an important and conserved oxygen-sensing mechanism depends on the oxygen-dependent activity of a family of Fe(II)-dependent thiol dioxygenases known as PLANT CYSTEINE OXIDASE (PCO) enzymes [45,46], which oxidize the free thiol groups of cysteine residues into sulfinic acid [45], and has structural similarities (but also differences) with other thiol dioxygenases [47]. An important feature of PCO enzymes in oxygen sensing is the dependency of their activity on oxygen levels [48] and the fact that the expression of some of the *Arabidopsis* PCOs is hypoxia dependent, while other family members are expressed constitutively [46]. Although the full complement of proteins modified by PCO enzymes is still unknown, several substrates with diverse physiological and developmental roles have been identified (e.g., VERNALIZATION2 (VRN2) which is involved in the control of flowering and LITTLE ZIPPER2 (ZPR2) in the shoot apical meristem [5,49]), including a set of conserved transcription factors that act as master regulators of hypoxia response in plants (Figure 1). These belong to the group VII ETHYLENE RESPONSE FACTOR (ERF-VII) family and have been shown to be substrates of PCOs both in vitro and in planta [45,46]. An important common feature of many ERF-VII transcription factors (e.g., *Arabidopsis* RELATED TO APETALA2.2 (RAP2.2), RAP2.3, RAP2.12, HYPOXIA RESPONSIVE ERF1 (HRE1), and HRE2) is the presence of a cysteine residue at position 2 (i.e., most ERF-VIIs start with the Met-Cys sequence) [50,51,52]. However, this Cys residue becomes N-terminal following removal of the initial Met residue by methionine aminopeptidases and can then be oxidized into Cys sulfinic acid by PCO enzymes [45,46]. Based on genetic evidence, as well as in vitro biochemical assays, the degradation of ERF-VIIs downstream of PCO modification has been shown to require conjugation of arginine (i.e., arginylation) by arginyl-transferases (ATE), followed by recognition and ubiquitination by the E3 ubiquitin ligase PROTEOLYSIS6 (PRT6) [45,46,50,51] (Figure 1). Both ATE enzymes and PRT6 are enzymatic components of the N-degron pathway, which targets its substrates for degradation depending on the nature of their N-terminal residue [53,54,55,56,57]. Under hypoxic conditions, PCO-mediated oxidation of the N-terminal Cys residue of ERF-VII transcription factors is limited due to decreased oxygen availability so that N-degron-dependent degradation is hindered [50]. As a result, ERF-VIIs accumulate in the cell and also translocate to the nucleus and regulate the expression of hypoxia-response genes [51,58] (Figure 1).

Notably, the N-degron pathway and its associated enzymatic components (ATE enzymes, as well as E3 ligases) are conserved in eukaryotes, including mammals (reviewed in [59]) (Figure 1). ATEs and N-degron pathway E3 ligases have been known for decades in mammals (these E3s are termed UBR proteins and include the partially functionally redundant UBR1, UBR2, UBR4, and UBR5 (reviewed in [60]), a mammalian equivalent of PCOs has only recently been identified and is known as the enzyme cysteamine (2-aminoethanediol) oxygenase (ADO) [61] (Figure 1). ADO also oxidizes the N-terminal cysteine of its protein substrates in an oxygen-dependent manner. Similar to PCOs, this can serve as a signal for arginylation and degradation by the N-degron pathway. Proteins which are degraded through this ADO-mediated and oxygen-dependent mechanism include the pro-inflammatory cytokine IL-32 [61], as well as regulator of G protein signalling 4 (RGS4) and RGS5 [61,62,63] (Figure 1). The latter have been previously shown to be involved in angiogenesis and the cardiovascular system which contributes to alleviating oxygen deficiency by promoting the growth of new blood vessels. Similarly to PCOs, the full complement of ADO substrates is not known, so that additional (still unknown) substrates may also play a role in the response of animals to hypoxia response.

A major mechanism by which mammals respond to hypoxia is through the degradation of the alpha subunit (HIF1α) of the heterodimeric transcription factor Hypoxia-Inducible Factor (HIF; the second subunit is known as HIF1β and is constitutively expressed). Similarly to plant ERF-VIIs, HIF1α is unstable under normoxia [64,65,66] due to the activity of oxygen-dependent Fe(II), 2-oxoglutarate enzymes, including prolyl hydroxylases (PHDs) [67,68] and factor inhibiting HIF (FIH) enzymes [69,70,71,72] (Figure 1). Specifically, in normoxia, two proline residues of HIF1α are hydroxylated by PHDs in an oxygen-dependent reaction. PHD-dependent HIF1α hydroxylation serves as a degradation signal that is recognized/bound by the von Hippel-Lindau (VHL) protein as part of the multisubunit cullin2/elongin-based E3 ligase and subsequently results in its ubiquitination and degradation [65,66,67,68] (Figure 1). In addition, oxygen-dependent FIH hydroxylates an asparagine residue of HIF1α, this time hindering the recruitment of transcriptional co-activators by HIF1 [69,70]. During hypoxia, PHD-mediated hydroxylation of HIF1α does not occur, resulting in HIF1α stabilization, as well as translocation to the nucleus and regulation of its target genes. The ubiquitin/proteasome-mediated regulation of HIF1α is hence conceptually very similar to that of the ERF-VII transcription factors in plants.

However, the regulation of HIF1α under hypoxia is more complex as it is, in fact, the target of many post-translational modifications [21]. Among these other post-translational modifications, the sumoylation of HIF1α contributes to its ubiquitin-dependent stability, but the mechanisms underlying the role of SUMO remain somewhat unclear, as opposite models have been proposed (Figure 1). Differences may be due to cell types and promoters used to drive the expression of SUMO in transfection assays, but also to the genetic backgrounds used, which may allow desumoylation of proteins or not [73]. On the one hand, it has been shown that hypoxia-induced sumoylation of HIF1α increases its stability and enhances its transcriptional activity [74,75]. This sumoylation and HIF1α stabilization have been shown to be facilitated by the RSUME (RWD-containing SUMoylation Enhancer) protein [75], which interacts with Ubc9, the SUMO E2 [75]. On the other hand, it has been shown that hypoxia up-regulates the SUMO protease SENP1 (Sentrin-specific protease 1), resulting in the desumoylation of HIF1α under hypoxia. In this case, SENP1 appears to stabilize HIF1α and increase its transcriptional activity [73], indicating that sumoylation, in fact, may negatively regulate HIF1α activity under hypoxia. In this model, sumoylation of HIF1α increases the affinity of VHL for its substrate, and hence SUMO positively contributes to HIF1α degradation by VHL [73]. In contrast, a more recent study found that the SUMO proteases SENP1 and SENP3 are inhibited under hypoxia in a reversible manner [25]. Yet another observation suggests that the sumoylation of HIF1α downregulates its transcriptional activity, possibly without altering its stability [76]. The controversy of how HIF1α is regulated by sumoylation extends to the SUMO E3 ligases that interact with HIF1α, with at least three having been identified [77,78,79]. Interestingly, many of the proteins that regulate HIF1α stability are themselves repressed by sumoylation under hypoxia (Figure 1), including VHL [80,81] and PHDs [82], hence highlighting the tight connections between the ubiquitin and SUMO systems in the regulation of hypoxia response mechanisms.

In contrast to the prominent role of sumoylation in the regulation of HIF1α activity, so far, very little is known about the potential role of SUMO in the regulation of hypoxia sensing or signal transduction in plants. In recent years, SUMO biology and the roles of sumoylation in plant development and in stress response have emerged (reviewed in [22]). Yet, ERF-VIIs or other known N-degron pathway enzymatic components are thus far not known to be sumoylated. Interestingly though, in plants, an alternative mechanism for the regulation of ERF-VIIs involves another set of E3 ubiquitin ligases than the N-degron-associated PRT6. These E3 ligases are SEVEN IN ABSENTIA OF *Arabidopsis thaliana* 1 (SINAT1) and SINAT2. Specifically, in *Arabidopsis*, simultaneous down-regulation of the functionally redundant SINAT1 and SINAT2 led to an increased accumulation of RAP2.12 (Figure 1). A similar effect of reduced SINAT1/2 transcription was observed when N-terminally HA-tagged RAP2.12 that is protected from N-degron-mediated degradation was expressed. This suggests that SINAT1/2 contribute to RAP2.12 degradation, likely independently of the N-degron pathway [83]. Because experiments were conducted under normal oxygen conditions, it is not clear whether the SINAT1/2-mediated destabilization of RAP2.12 relates to its roles in the regulation of hypoxia response. Indeed, in recent years, ERF-VIIs have been linked to the regulation of plant response to multiple abiotic and biotic stresses [83,84,85,86,87]. RAP2.2 was also found to interact with SINAT2 [88], but it remains to be determined if its stability is regulated by this E3 ubiquitin ligase. Similarly, SINAT E3 ligases have been shown to regulate a wide range of processes in *Arabidopsis*, including development responses to abiotic and biotic stresses (reviewed in [89]), as well as autophagy-related processes [90,91,92]. Strikingly, human homologs of SINAT E3 ligases, Seven in absentia homolog 1a (SIAH1a) and SIAH2, also regulate hypoxia response (e.g., [93,94,95]). Important substrates of SIAH1a and SIAH2 are PHD1 and PHD3 (Figure 1), which hydroxylate HIF1α [96]. Notably, SIAH2-mediated degradation of PHD3 is enhanced under hypoxic conditions, in part as a result of SIAH2 increased expression upon hypoxia [96]. Nakayama et al. (2004) suggest that SIAH2-mediated degradation of PHD3 forms a positive feedback mechanism to further increase HIF1 levels and the induction of the hypoxia response program [96]. Interestingly, the fact that PHD2 is not targeted for degradation by SIAH2 contributes to the different functions of the PHD proteins, with PHD3 playing a more important role under hypoxia, while PHD2 may play a more important role under normoxia [96]. The latter is akin to the potential sub-functionalization of PCOs in *Arabidopsis* based on their differential expression. For example, PCO4/5 are constitutively expressed, while PCO1/2 expression is induced upon hypoxia [46]. One possibility is that PCO4/5 play a role in controlling ERF-VII protein levels under normoxia, while PCO1/2 may play a role upon re-oxygenation [48]. In summary, SEVEN IN ABSENTIA E3 ligase families may have a functionally conserved role in the regulation of hypoxia response in plants and mammals. The comparison of their roles in the regulation of oxygen sensors and their target substrates brings to mind unresolved questions in the plant field. For example, how are PCO protein levels regulated?

## 3. Ubiquitin and Sumoylation Function in Sugar/Energy Sensing and Downstream Signal Transduction

Survival under hypoxia requires mechanisms to detect and respond to the ensuing energy and carbohydrate crisis. The kinase SNF1-RELATED KINASE1 (SnRK1) in plants and its ortholog AMP-ACTIVATED PROTEIN KINASE (AMPK) in mammals [97] play an essential role in this context by regulating transcriptional and translational changes, as well as metabolic activities during the energy and carbohydrate crises that can occur during hypoxia stress (reviewed in [98,99,100]). These kinases are heterotrimeric complexes that include an α catalytic subunit and two regulatory subunits (β and γ). The α catalytic subunits have a conserved kinase domain at their N-terminus, including the so-called T-loop whose phosphorylation in mammalian α subunits is important for AMPK activation [101,102]. In contrast, the role of T-loop phosphorylation in the activation of plant α subunits is more contentious in the absence of a clear correlation between T-loop phosphorylation and catalytic activity ([103,104]). Both SnRK1 and AMPK encompass a C-terminal ubiquitin-associated (UBA) domain, which is typically associated with mediating interaction with ubiquitinated proteins. However, the C-terminal domain of the α subunit of AMPK is considered as an autoinhibitory domain [98], while in *Arabidopsis*, the UBA domain appears to play a role in SnRK1 activation [105].

In *Arabidopsis*, the two catalytic α subunits encoded by AKIN10 (SnRK1.1 or SnRKα1) and AKIN11 (SnRK1.2 or SnRKα2) have been shown to be particularly relevant in the context of hypoxia and sugar depletion [103,106,107,108]. Seminal work by Baena-Gonzalez et al. (2007) showed that AKIN10 and AKIN11 contribute to regulating the transcription of over 1000 genes [103], including the activation of diverse catabolic pathways that provide alternative sources of energy (e.g., starch, sucrose, protein, or lipid degradation). At the same time, AKIN10 contributes to the repression of genes associated with anabolic pathways or energy-consuming processes such as ribosome biogenesis. Although the results above were not obtained under hypoxic conditions, the AKIN10-regulated processes identified are directly relevant to hypoxia stress and associated energy crisis. In parallel, several independent results indicate a direct involvement of SnRK1 in mediating plant responses to hypoxia. For example, the expression of *DARK-INDUCED6* (*DIN6*), an AKIN10-response marker gene, is up-regulated under hypoxic conditions [103,106]. Similar results were found with rice OsSnRK1.1, suggesting a conserved role of SnRK1 kinases in plants in response to hypoxia [106]. Additional experiments showed that the expression of core hypoxia-response genes, such as *ALCOHOL DEHYDROGENASE1* (*ADH1*) and *PYRUVATE DECARBOXYLASE* (*PDC*), could be induced by SnRK1, possibly through a mechanism that involves the recruitment of AKIN10 to the promoter region of these genes [106,109]. Finally, a phosphoproteomic analysis of proteins obtained upon submergence in the dark further revealed putative targets of SnRK1 in *Arabidopsis*, as well as roles in the regulation of MAP kinase signaling and in the control of translation during low oxygen conditions [107,110].

Important questions relate to the regulation of SnRK1, and in particular (i) its activation upon hypoxia/low energy, and/or (ii) its repression (e.g., upon return to normoxia). In contrast to our understanding of mammalian AMPK activation by high AMP/ATP or high ADP/ATP ratios (reviewed in [100]), plant SnRK1 activation upon low energy levels appears to be via a different mechanism, which remains to be elucidated in detail [98,111] but appears to require SnRK1 ACTIVATING KINASE1 (SnAK1; also termed GEMINIVIRUS REP INTERACTING KINASE2, GRIK2) and SnAK2 (or GRIK1) [112,113]. Interestingly, SnAK1/2 are homologs of mammalian Liver Kinase B1 (LKB1), which is also a known kinase activator of AMPK [114], thus highlighting a direct parallel between the regulation of SnRK1/AMPK in plants and mammals. In addition, in vitro studies suggest that SnRK1 activation may also depend on the redox status in the cell [115].

SnRK1 is inhibited indirectly by micromolar levels of trehalose-6-phosphate (T6P) [116,117], a sugar that acts as an indicator of sucrose availability and plays important regulatory roles during development as well as in response to stresses (reviewed in [118]). In the context of hypoxia, the resulting lower T6P levels [107] likely contribute to the activation of SnRK1 activity. The role of T6P in SnRK1 regulation is particularly interesting considering that SnRK1 itself phosphorylates T6P synthases (TPS) [107,119,120,121] and regulates the expression of TPS-coding genes [103].

The ubiquitin/proteasome system is another important regulator of SnRK1 activity. For example, AKIN10 mutant proteins that are either inactive or carry a point mutation in the T-loop accumulate to higher levels than wild-type AKIN10 proteins [103]. This suggests that activated AKIN10 or SnRK1 complex may correlate with instability or degradation of the catalytic subunit [122]. In yeast-2-hybrid and GST pulldown experiments, AKIN10 interacts with components of the ubiquitin/proteasome system, including ASK1 (a subunit of cullin1-based SCF E3 ubiquitin ligases) and α1/PAD1, a subunit of the 26S proteasome [123]. However, it remains unclear whether an SCF-type ubiquitin ligase contributes to the degradation of AKIN10. Instead, the interaction between ASK1 and AKIN10 is mutually exclusive of the interaction between AKIN10 and PLEIOTROPIC REGULATORY LOCUS1 (PRL1), which acts as a negative regulator of SnRK1 in vitro [124]. Notably, PRL1 comprises a DWD motif (within its WD40 domain), which is important for the formation of DDB1-CUL4-ROC1 ubiquitin ligase complexes, in which DWD-containing proteins act as substrate recognition subunits [125]. The PRL1/AKIN10 interaction hence suggests that PRL1 may act to target SnRK1 for degradation in a DDB1-CUL4-ROC1-dependent manner. In agreement with this idea, AKIN10 protein levels are higher in *prl1* or *cul4 Arabidopsis* mutant plants, and in cell-free degradation assays, AKIN10 degradation appears to require PRL1 and CUL4 [125]. The same mechanism likely applies to AKIN11, as this subunit has also been found to interact with PRL1 [124]. Another WD40 domain protein, 5PTase13 (myoinositol polyphosphate 5-phosphatase), also interacts with AKIN10 and regulates SnRK1 activity depending on sugar availability [126]. Briefly, 5PTase acts as a negative regulator of SnRK1 under no nutrient conditions but as a positive regulator under low nutrient conditions [126]. It has been proposed that PRL1 and 5PTase13 may have opposite roles in the regulation of SnRK1 activity with the interaction of AKIN10/11 with 5PTase13 under low nutrient conditions protecting AKIN10/11 from its PRL1-mediated degradation [126]. However, experimental evidence to support this model is still needed. Notably too, while roles of the ubiquitin/proteasome system in the regulation of SnRK1 activity/stability are established, how they apply specifically in the context of hypoxia stress and of re-oxygenation is, to the best of our knowledge, not clearly understood.

In mammals, AMPK is also regulated via ubiquitination and proteasomal degradation through mechanisms that involve several E3 ubiquitin ligases and ubiquitin chains (e.g., K29, K63, and K48 [127,128,129,130]), depending on the organ or tissue, or physiological conditions. For a more comprehensive review on this topic, we refer the reader to [131]. Here, we will instead focus on selected examples that illustrate the opposite roles played by the ubiquitin/proteasome system in the regulation of AMPK. For example, AMPKα ubiquitination via K63 chains decreases its interaction and T-loop phosphorylation by LKB1 without affecting the levels of AMPKα [132]. In addition, the USP10 deubiquitinase, which is phosphorylated and further activated by AMPKα, deubiquitinates AMPKα resulting in a stronger interaction with LKB1 and activation of AMPKα [132]. Hence, USP10-mediated deubiquitination of AMPKα appears to be part of a positive feed-forward loop to amplify AMPKα activation [132] and counteracts the negative effect of the K63 ubiquitin chains that hinder AMPK activation by LKB1. This particular example highlights the role of deubiquitinases in the activation of AMPK but also highlights that potential roles of deubiquitinases in the regulation of SnRK1 in plants also need to be explored. The second example of interest for a comparison of SnRK1/AMPK is that of the role of DDB1-CUL4-ROC1 E3 ubiquitin ligases associated with the substrate adaptor Cereblon (CRBN) in the ubiquitination and proteasomal degradation of the regulatory AMPKγ subunit [133]. Indeed, DDB1-CUL4-ROC1 E3 ubiquitin ligases are also involved in the regulation of SnRK1 but with known effects on SnRK1α instead as. In contrast to mammals, in plants, less is known about the regulation by the ubiquitin/proteasome of non-catalytic subunits of SnRK1. Finally, in skeletal muscle cells, high-glucose conditions trigger the interaction of AMPKα2 with the ubiquitin ligase WWP1. This is followed by AMPKα2 ubiquitination and degradation by the 26S proteasome, thus providing a mechanism to negatively regulate AMPK activity upon return to high energy status [134]. This last example highlights some of the roles of the ubiquitin/proteasome system in regulating AMPK function upon return to higher energy levels (e.g., similar to re-oxygenation), an aspect that is, to the best of our knowledge, under-explored in the plant field [135].

In the case of SnRK1/AMPK as well, SUMO and ubiquitin conjugation appear to jointly regulate the activity of these kinases. SnRK1α1 (i.e., AKIN10) is sumoylated in a AtSIZ1 (SAP AND MIZ1 DOMAIN- CONTAINING LIGASE1; one of the E3 SUMO ligases in *Arabidopsis*) dependent manner [24,136]. Sumoylation was shown to be necessary for SnRK1α1 ubiquitination and degradation in a proteasome-dependent manner [24]. However, the E3 ubiquitin ligase responsible for SnRK1α1 ubiquitination following sumoylation remains to be identified. Possible candidates include PRL1-DDB1-CUL4-ROC1, but also E3 ubiquitin ligases that are known to recognize sumoylated proteins for ubiquitination and degradation, such as SUMO-targeted ubiquitin ligases (StUbLs), five of which have been identified in *Arabidopsis* [24,41]. Because these results were obtained under normoxic conditions, an important question that remains is whether these regulatory mechanisms apply in the context of hypoxia stress. In mammals, SUMO is also involved in the regulation of specific AMPK subunits, with opposite roles being described in the literature. In contrast with the role of sumoylation in plants, SUMO conjugation to AMPKβ2 instead contributes to increasing AMPK activity by protecting AMPKβ2 from degradation by the ubiquitin/proteasome system [137]. In addition, AMPKα1 and α2 are also sumoylated, resulting in a decreased AMPK activity without altering the abundance (and hence likely their stability) of these proteins [138].

In summary, there are striking parallels between the regulatory mechanisms of SnRK1 and AMPK in plants and mammals, with orthologous regulators being involved in both (e.g., SnAKs and LKB1), or similar types of E3 ubiquitin ligases (e.g., CUL4-based E3s). However, the parallels also reveal gaps of knowledge in understanding the role of the ubiquitin and SUMO systems in the context of re-oxygenation or in the regulation of non-catalytic subunits of the SnRK1 complex.

## 4. Ubiquitin and SUMO in the Regulation of NO Signalling during Hypoxia

As indicated above, NO acts as a key signaling molecule in a multitude of developmental as well as stress pathways in plants and mammals, including hypoxia [17,139]. This reactive gaseous molecule plays roles in the sensing of oxygen/hypoxia and downstream signaling events. For example, NO production in roots is an important component of hypoxia tolerance, as the application of NO scavengers reduced plant survival to hypoxic stress [140]. This beneficial effect stems from a range of NO-mediated effects, including the induction of enzymes such as ADH1 and PDC [140,141,142], as well as the regulation of genes necessary to shift ATP generation to oxygen-independent mechanisms and reduce oxidative stress in response to low oxygen [142,143]. In mammals, NO also contributes to the regulation of oxygen sensing mechanisms (see below) while also facilitating vasodilation, anti-thrombotic effects, and angiogenesis to increase oxygenated blood getting to hypoxic tissues [144,145,146]. The latter effect is similar to the role of NO in plants in facilitating the transport of oxygen to hypoxic tissues through the formation of aerenchyma, which facilitates gas exchange in waterlogged plants [147].

### 4.1. NO Production and Regulation

**Figure 2:**
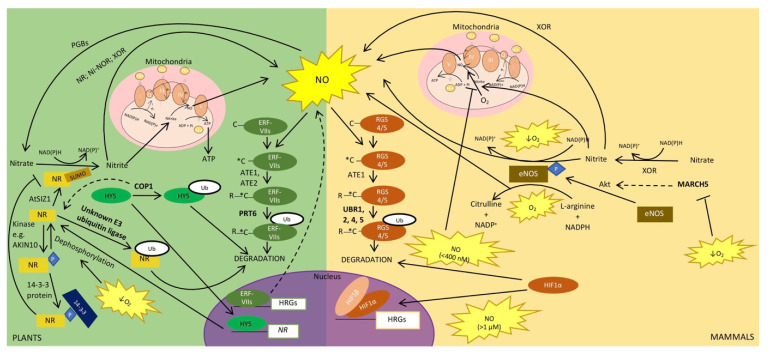
Regulation of NO production and effects of NO on hypoxia sensing and response. In plants, NR catalyzes the first step of NO biosynthesis turning nitrate into nitrite. Nitrite can then be reduced to NO by NR, NI-NOR, XOR, and complexes III and IV of the mitochondrial electron transport chain. The latter results from the use of nitrite as a terminal electron acceptor (i.e., the nitrite-phytoglobin (PGB) cycle). In mammals, eNOS produces NO *via* oxidative or reductive mechanisms depending on the oxygen level. XOR is also capable of reducing nitrite to NO, as is complex III of the mitochondrial electron transport chain. NR and eNOS activity and their stability are regulated directly and indirectly by phosphorylation and ubiquitination. NR is also regulated by SUMO. In both plants and mammals, the N-degron pathway acts as a sensor of NO. In mammals, NO also affects the HIF-dependent oxygen-sensing pathway. Solid lines: confirmed pathways/interactions; dashed lines: proposed pathways/interactions.

NO production rapidly increases in response to hypoxia in both plants and mammals [140,148,149,150,151] and can be produced by oxidative as well as reductive pathways [17,152,153,154]. In this review, we focus on the main NO generating pathways in plants and mammals, as well as the pathways they have in common. For more comprehensive reviews of NO production, we refer the readers to these reviews: [17,153,154,155]. In plants, the main source of NO originates from the reductive pathway. This involves the reduction of nitrate to nitrite and then of nitrite to NO in the presence of NAD(P)H [156]. Nitrate reductase (NR), an enzyme previously shown to be important for plant survival under hypoxia [157], is involved in the first step of this NO biosynthesis pathway (Figure 2). In many plant species, there are two isoforms of NRs. In *Arabidopsis* these are encoded by the genes *NIA1* and *NIA2*. The NIA1 and NIA2 proteins both form homodimers but differ in their activity. For example, NIA2 catalyzes nitrate reduction more efficiently than NIA1. After the generation of nitrite by NR, the subsequent reduction of nitrite to NO is also mediated by NR. In this case, NIA1 is a more efficient nitrite reductase compared to NIA2 [158]. Nitrite reduction is also performed by a root plasma membrane-bound protein (Figure 2), which is referred to as nitrite-NO reductase (NI-NOR) [159]. Nitrite can also be reduced by complexes III and IV of the mitochondrial electron transport chain; however, this only occurs in roots under hypoxic conditions (e.g., soil waterlogging) [156,160] (Figure 2). Specifically, in response to low oxygen conditions, root mitochondria and NRs form an ATP-generating cycle known as the nitrite-phytoglobin cycle. As part of this cycle, NR produces nitrite for use as a terminal electron acceptor for the electron transport chain in mitochondria, replacing oxygen. This results in the generation of ATP and NO [160]. The resulting NO is scavenged by phytoglobins in order to maintain NO levels and regenerate nitrate to be used by NR, completing the cycle [143]. The nitrite-phytoglobin cycle produces ATP at the same rate as glycolysis and also regenerates NAD(P)+, suggesting that it could act as an alternative anaerobic pathway to fermentation in plants [143,160].

In mammals, when oxygen is available, NO is produced via the oxidation of L-arginine in the presence of multiple co-factors and NADPH to form NO and citrulline. Nitric oxide synthases (NOSs) are particularly important for this NO biosynthesis pathway (Figure 2). There are three isoforms in mammals, endothelial (eNOS), neuronal (nNOS), and inducible (iNOS), all of which function as homodimers in order to produce NO in different cell types and in response to different conditions (reviewed in [144]). In contrast, the presence of NOS in plants is still debated and remains an important open question. While NOS-like activity has been detected in plants [144], the specific protein(s) which mediates plant NOS activity has yet to be isolated, and analyses of plant genomes have so far not led to the identification of plant homologs to the mammalian NOSs [17,153]. While all three mammalian NOS isoforms have important physiological roles in both homeostasis as well as stress conditions, of particular importance in mammalian tolerance to hypoxia is the constitutively expressed eNOS. NO production by eNOS (especially in vascular endothelial cells) during hypoxia and anoxia allows vasodilation, angiogenesis, and wound repair [144,146,149]. It has also been shown clinically that eNOS has protective effects in ischaemic brain injury and stroke [150]. Oxidation of L-arginine to NO by eNOS requires oxygen. Depending on the severity of hypoxia, there may be sufficient oxygen to allow eNOS to continue to oxidize L-arginine to NO. However, in acute hypoxia and anoxia, eNOS has nitrite reductase activity and can reduce nitrite into NO [149,151] (Figure 2). This dual activity probably explains why eNOS was found to be the only NOS isoform capable of significant NO production from normoxia to anoxia [150]. Xanthine oxidoreductase (XOR) has also been found to have nitrite reductase activity in mammals [150,151,152] (Figure 2). Furthermore, XOR is capable of reducing nitrate to nitrite in normoxic and hypoxic conditions [152]. Interestingly, plants also have a xanthine oxidoreductase found in the peroxisome, which is capable of reducing nitrite to NO during hypoxia [17]. Lastly, as in plants, complex III of the mammalian mitochondrial electron transport chain produces NO through reduction of nitrite in hypoxia [161] (Figure 2). In sum, apart from the absence of known NOSs in plants so far, direct parallels can be drawn for other NO-producing pathways in plants and mammals.

### 4.2. Plant NRs and Mammalian eNOS Are Regulated by Sumoylation and Ubiquitination

Phosphorylation of plant NRs leads to 14-3-3 protein binding and inhibition of NR activity [157]. In keeping with the fact that NR has increased activity in hypoxia, NR dephosphorylation and release of the 14-3-3-bound protein was observed in anoxic roots of tomato plants [157] (Figure 2). *Arabidopsis* NIA1 and NIA2 proteins have also been shown to undergo sumoylation by the E3 SUMO ligase, AtSIZ1, with this sumoylation resulting in increased NR activity [23]. Whether it also increases nitrite reductase activity remains an open question, although the finding that *siz1-2* mutants have decreased NO production suggests that sumoylation may also regulate the nitrite reductase activity of NRs [23]. Because the study of NR regulation by SUMO was conducted under normoxia, another question that remains to be answered is whether sumoylation of NR enzymes has a role in regulating NR activity during hypoxia. A later study [162] also suggests that sumoylation of NIA1 and NIA2 by AtSIZ1 correlates with their localization to the nucleus, but the role of NR in the nucleus remains to be investigated, especially under hypoxic conditions.

Ubiquitin conjugation to NRs also regulates their activity in *Arabidopsis* (Figure 2). Specifically, ubiquitin was found to modulate the abundance of NIA1 and NIA2 through a mechanism that requires the activity of the E3 ubiquitin ligase CONSTITUTIVE PHOTOMORPHOGENIC1 (COP1) [162]. However, in a yeast two-hybrid screen, COP1 and *Arabidopsis* NRs do not interact with each other, suggesting that other proteins, co-factors, or post-translational modifications not found in yeast might be necessary for interaction between COP1 and *Arabidopsis* NR proteins [162]. Alternatively, another E3 ubiquitin ligase could be responsible for NR degradation. The effects of COP1 on NR activity could also be indirect. Indeed, the transcription factor LONG HYPOCOTYL5 (HY5) is a known target of COP1 that induces NR gene expression and degradation of HY5 by COP1 results in reduced NR protein levels [163]. COP1 has been associated with differences in flooding survival strategies in Rumex species with up-regulation of *COP1* upon submergence in *Rumex palustris*, but not in *R. acetosa* [164]. COP1′s role in hypoxia must be further elucidated, in particular, whether its role in regulating NR activity in normoxia also extends to hypoxic conditions and, if so, whether NR regulation by COP1 under submergence contributes to survival strategies.

Similarly, in mammals, ubiquitination is important in regulating eNOS in hypoxic conditions (Figure 2). The E3 ubiquitin ligase Membrane Associated Ring-CH-type finger 5 (MARCH5) has a protective role against the effects of hypoxia in endothelial cells [146]. Specifically, MARCH5 increases eNOS expression at the transcriptional and translational levels. Interestingly, MARCH5 also increases the expression of the protein kinase Akt. This, together with the use of Akt inhibitors, has suggested that Akt may be the kinase that is responsible for phosphorylating and activating eNOS. Hence, MARCH5 induces its protective effect on endothelial cells in hypoxia by inducing NO production through Akt-dependent eNOS phosphorylation [146].

### 4.3. Regulation of NO and Oxygen Sensing Pathways

NO plays a central role in the regulation of the oxygen-sensing pathways in plants and mammals. As well as sensing oxygen, the N-degron pathway also serves as a NO sensor (Figure 2), with oxidation of N-terminal cysteine residues of N-degron pathway substrates occurring in the presence of NO [62,148]. It has been suggested that the N-degron pathway initially evolved to sense NO, but with increasing environmental oxygen, due to the evolution of photosynthetic organisms, sensing of these two gases was joined and conducted by the same pathway [1]. In plants, NO and oxygen are required for N-terminal cysteine oxidation through the action of the PCOs [52]. It was thought that NO sensing by the N-degron pathway in mammals occurred through the S-nitrosylation of the N-terminal cysteine, which can be further oxidized to Cys sulfinic and Cys sulfonic acids [62]. This was suggested prior to the discovery of ADO in mammals [61]. Whether NO is involved in the non-enzymatic oxidation of N-degron pathway substrates or whether NO oxidizes these substrates in conjunction with oxygen and the cysteine oxidase, ADO, will need to be explored. This NO-dependent oxidation of N-terminal cysteines on N-degron substrates (e.g., RGS4 and RGS5 in mammals [62] and ERF-VIIs in plants [148]) results in their degradation through the N-degron pathway, as outlined above.

In plants, a depletion of NO prior to the onset of hypoxia could prime plant responses to this stress and increase their survival [165]. This initial depletion of NO is likely the result of elevated ethylene levels in submerged tissue, which induces the up-regulation of *PHYTOGLOBIN1* (*PGB1;* also known as *HEMOGLOBIN1* (*HB1*)) [165], a known scavenger of NO during hypoxia [166,167] (Figure 2). Up-regulation of *PGB1* allows the stabilization of the ERF-VII transcription factors, thus promoting hypoxia tolerance [165]. Notably, the stabilization of N-degron pathway substrates, including the ERF-VII transcription factors, leads to NO induction. Hence, the early suppression of NO at the onset of hypoxia not only allows stabilization of the ERF-VIIs in order to drive hypoxia response genes but may also contribute to inducing the NO burst in response to hypoxia [148].

Similarly to the regulation of ERF-VIIs by NO, the regulation of the mammalian HIF1-dependent oxygen-sensing pathway by NO has also been proposed during hypoxia, although this remains controversial. Indeed, reports have shown conflicting roles of NO on HIF1 activity, with studies showing both positive [168] as well as negative [169,170] effects on HIF1α accumulation. Mateo et al. [171] put forth that these differences are a result of NO concentration within the cell (Figure 2). High concentrations of NO (>1 μM) had a positive effect on HIF1α stability, while low NO concentrations (<400 nM) destabilized HIF1α. This concentration-dependent effect of NO on HIF1α stabilization occurs in high (21%) as well as low oxygen (3%) conditions. It was also shown that the destabilizing effect of low NO concentrations on HIF1α during hypoxia was dependent on the mitochondrial electron transport chain, while the stabilizing effect of high NO concentrations was not [169,170,171]. The repression of HIF1α was proposed to stem from the redistribution of the limited oxygen within the cell from the mitochondrial electron transport chain to be used for other oxygen-dependent processes. Interestingly, this inhibition of mitochondrial complexes by NO in order to redistribute oxygen for other oxygen-dependent processes as well as reducing the rate of oxygen consumption has also been found in plants [143].

### 4.4. Downstream Effects of NO also Require the Ubiquitin System and Ubiquitin-Like Proteins

The effects of NO are also mediated by its post-translational modification of proteins. In both plants and mammals, the NO burst during hypoxia coincides with an increase in a NO-based post-translational modification known as protein S-nitrosylation [141,172]. The levels of S-nitrosoglutathione (GSNO), a stable source of intracellular NO, mediate protein S-nitrosylation (Figure 2). The increase in S-nitrosylation during hypoxia was shown to stem from the autophagic degradation of the inhibitor of GSNO, GSNO REDUCTASE1 (GSNOR1) in plants [141]. Mammalian GSNOR is conserved for the ATG8 recognition site and amino acid residue for S-nitrosylation, which suggests that a similar S-nitrosylation-dependent mechanism for the regulation of GSNOR during hypoxia may exist in mammals [141]. In the context of other stresses, protein S-nitrosylation has been shown to contribute to the regulation of protein targets by the ubiquitin/proteasome (e.g., [173,174]). However, systematic studies on the connection between protein S-nitrosylation during hypoxia and their stability remain to be carried out. While not explored in the context of hypoxia specifically, the SUMO-conjugating enzyme SCE1 has been shown to be regulated by S-nitrosylation [175]. It will be interesting to determine if such regulatory mechanisms also exist in the context of hypoxia and whether these serve as a link between the SUMO and ubiquitin systems.

In sum, in both mammals and plants, NO plays a central role in the regulation of responses to hypoxia. The comparison highlights that a full understanding of the role of NO in hypoxia will require further studies into the spatial, temporal, and concentration dynamics of NO in the cells and tissues of plants and mammals. In plants, many of the mechanisms that regulate NO production or scavenging remain to be studied specifically under hypoxic conditions.

## 5. Conclusions

Oxygen sensing, as well as several of the signaling events that occur downstream and govern the onset of hypoxia response, are conceptually similar in plants and mammals, even though the proteins involved are not always homologous (e.g., regulation of ERF-VIIs and HIF1). In other aspects, homologous components (e.g., SnRKs and AMPK, as well as their respective activating kinases) are involved in downstream signaling pathways that mediate the response to hypoxia. A conserved feature of the regulation of oxygen sensing and downstream signaling pathways is in the central role that the ubiquitin/proteasome system plays, often combined with the role of other ubiquitin-like proteins such as SUMO. In plants, in particular, the role of SUMO in the regulation of oxygen sensing and hypoxia response remains under-explored. Another area that requires more specific studies is that of re-oxygenation and return to normoxia. In the context of waterlogging or flooding, re-oxygenation is known to be an important stress when water recedes. Yet, less is known about the mechanisms underlying a return to homeostasis. Additional aspects, such as a better spatial and temporal resolution for the different mechanisms, also need to be addressed, perhaps in relation to different levels of oxygen or signaling molecules (e.g., NO) as well. In the context of ubiquitin-like proteins, another important emerging player is autophagy [176,177], which not only relies on the conjugation of ubiquitin to targets but also ubiquitin-like proteins, such as ATG8.

## Data Availability

Not applicable.
